# Risk Factors and Screening for *Trypanosoma cruzi* Infection of Dutch Blood Donors

**DOI:** 10.1371/journal.pone.0151038

**Published:** 2016-03-07

**Authors:** Ed Slot, Boris M. Hogema, Michel Molier, Aldert Bart, Hans L. Zaaijer

**Affiliations:** 1 Department of Blood-borne Infections, Sanquin Research, Sanquin Blood Supply Foundation, Amsterdam, the Netherlands; 2 Department of Virology, Sanquin Diagnostic Services, Sanquin Blood Supply Foundation, Amsterdam, the Netherlands; 3 Department of Medical Microbiology (CINIMA), Academic Medical Center, Amsterdam, the Netherlands; FDA, UNITED STATES

## Abstract

**Background:**

Blood donors unaware of *Trypanosoma cruzi* infection may donate infectious blood. Risk factors and the presence of *T*. *cruzi* antibodies in at-risk Dutch blood donors were studied to assess whether specific blood safety measures are warranted in the Netherlands.

**Methodology:**

Birth in a country endemic for Chagas disease (CEC), having a mother born in a CEC, or having resided for at least six continuous months in a CEC were considered risk factors for *T*. *cruzi* infection. From March through September 2013, risk factor questions were asked to all donors who volunteered to donate blood or blood components. Serum samples were collected from donors reporting one or more risk factors, and screened for IgG antibodies to *T*. *cruzi* by EIA.

**Results:**

Risk factors for *T*. *cruzi* infection were reported by 1,426 of 227,278 donors (0.6%). Testing 1,333 at-risk donors, none (0.0%; 95%, CI 0.0–0.4%) was seroreactive for IgG antibodies to *T*. *cruzi*. A total of 472 donors were born in a CEC; 553 donors reported their mother being born in a CEC; and 1,121 donors reported a long-term stay in a CEC. The vast majority of reported risk factors were related to Suriname and Brazil. Overall, the participants resided for 7,694 years in CECs, which equals 2.8 million overnight stays. Of those, 1.9 million nights were spent in Suriname.

**Conclusions/Significance:**

Asymptomatic *T*. *cruzi* infection appears to be extremely rare among Dutch blood donors. Blood safety interventions to mitigate the risk of *T*. *cruzi* transmission by transfusion would be highly cost-ineffective in the Netherlands, and are thus not required.

## Introduction

Chagas disease (or American trypanosomiasis) is a systemic, chronic disease caused by the protozoan parasite *Trypanosoma cruzi*, which is transmitted to humans on the Latin American continent by blood-feeding triatomine bugs followed by autoinoculation, through mother-to-child transmission, via transfusion and transplantation, and by ingestion of contaminated food [1,2]. The number of incident cases decreased from an estimated 700,000 new cases per year in 1990 to 41,200 in 2006 [3]. The current prevalence of *T*. *cruzi* infection is not well documented, but it is estimated that 6 to 8 million Latin Americans are chronically infected [4]. Most of them do not show clinical symptoms for a period of years or decades after being infected. During this period, a person can present to donate blood in seemingly good health, and subsequently may donate an infectious unit of blood.

Over the past decades millions of people migrated from Latin America to countries with sporadic or no vectorial transmission of *T*. *cruzi* [5], having a negative impact on transfusion and transplantation safety levels in at least some non-endemic countries [6–9]. In addition, there are concerns in non-endemic countries about the safety of blood collected from donors who visited Latin America, irrespective of the person’s country of origin. Targeted donor selection and screening policies have been adopted by blood transfusion services in non-endemic regions such as the United States, Canada, and several European countries [6,10–13]. So far, no specific measures for prevention of *T*. *cruzi* transmission by blood products have been taken in the Netherlands.

Data on the prevalence of subclinical Chagas infection among Latin American immigrants and their offspring in the Netherlands is lacking. It is also unknown how many Dutch blood donors were born in a country endemic for Chagas disease, or how many donors were born from mothers who had been born in those countries. Additionally, no data are available regarding long-term visits of Dutch blood donors to Latin America. To assess whether interventions to prevent *T*. *cruzi* transmission by blood products are warranted in the Netherlands, we prospectively studied risk factors for *T*. *cruzi* infection among Dutch blood donors, and the presence of antibodies to *T*. *cruzi* in at-risk donors.

## Materials and Methods

For this study countries with a *T*. *cruzi* prevalence in humans of at least 1%, as estimated by the Pan American Health Organization (PAHO) [14], were considered as countries endemic for Chagas disease (CEC); i.e. Argentina, Bolivia, Brazil, Ecuador, El Salvador, French Guiana, Guatemala, Guyana, Honduras, Mexico, Nicaragua, Paraguay, Suriname, and Venezuela. Birth in a CEC, having a mother who was born in a CEC, or having resided for a continuous period of at least six months in one or more CECs, were considered risk factors for *T*. *cruzi* infection.

In the Netherlands, blood and blood components are collected only from voluntary, non-remunerated repeat donors. From March through September 2013, additional questions were included in the routinely used donor health questionnaire to identify donors at risk of *T*. *cruzi* infection. All blood collection centers throughout the country participated. Donors who reported one or more risk factors were asked to participate in the study. Consenting donors were interviewed by qualified staff of the collection centers. Risk factor information was noted on a standardized form, and a serum sample was obtained as part of routine sample collection. The serum samples were screened for IgG antibodies to *T*. *cruzi* (EIA Test System, Ortho Clinical Diagnostics, Johnson & Johnson, Raritan, NJ), following the manufacturers instructions. The assay uses a heterogeneous mixture of antigens prepared from an epimastigote lysate of cultured *T*. *cruzi* parasites. The performance of the assay has previously been reported [10,15,16].

### Ethics Statement

Both data on risk factors for *T*. *cruzi* infection and serum samples were collected only from voluntary, non-remunerated repeat blood donors who provided written informed consent as part of routine donor selection and blood collection procedures. The study was reviewed and approved by the Ethical Advisory Council of Sanquin Blood Supply Foundation before the study began.

## Results

During the study period 227,278 donors were qualified to donate blood or blood components, representing 82.5% of all active donors in the Netherlands in 2013. All of these donors were questioned for Chagas risk factors; 1,434 donors (0.6%) indicated one or more risk factors. Of these, 101 donors (7.0%) were excluded from the study because of incomplete forms (n = 48), missing serum samples (n = 45), or erroneous risk factors (n = 8). Hence 1,333 donors were screened for IgG antibodies to *T*. *cruzi*.

Characteristics of the study population are provided in Table 1. Both sexes contributed equally and all age groups were well represented. Risk factors for *T*. *cruzi* infection were well distributed over the age groups (Table 2). The risk factors per country are specified in Table 3. A total of 472 donors were born in a CEC; 553 donors reported having a mother from such a country; and 1,121 donors reported a total of 1,185 long-term episodes in a CEC. The majority of participants born in a CEC originated from Suriname or Brazil (400/472; 84.7%); and 496 of 553 (89.7%) mothers were of Surinamese or Brazilian origin.

**Table 1:** Age and gender of Dutch blood donors who reported risk factors for *T*. *cruzi* infection and were tested for IgG antibodies to *T*. *cruzi*.

Age (years)	Female	Male	Total
18–29	193	105	298
30–39	155	102	257
40–49	136	138	274
50–59	110	171	281
60–69	72	151	223
Total	666	667	1,333

**Table 2:** Distribution of risk factors for *T*. *cruzi* infection over donor age groups in the Netherlands.

Age (years)	Donor born in an endemic country^a^	Mother born in an endemic country	Long-term stay^b^ in an endemic country	One or more of the risk factors
18–29	68	147	195	298
30–39	86	133	199	257
40–49	130	122	252	274
50–59	139	105	267	281
60–69	49	46	208	223
Total	472	553	1,121	1,333

^a^ Country-wide *T*. *cruzi* prevalence of at least 1%, according to PAHO [14]

^b^ Residence or travel for a continuous period of at least 6 months

**Table 3:** Dutch blood donors and risk factors for *T*. *cruzi* infection.

	Risk factor			Length of stay		
	(donor reports)			(months)		
	Donor born in	Mother born in	Long-term stay^a^	Total for all donors	Mean per donor	RI^b^ per year of residence (95% CI)
Argentina	8	15	50	2,835	57	0.0–2.0%
Bolivia	4	1	28	497	18	0.0–10.6%
Brazil	75	48	169	14,575	86	0.0–0.4%
Ecuador	6	6	33	1,764	53	0.0–3.2%
El Salvador	1	1	7	536	77	0.0–9.9%
French Guiana	0	0	3	91	30	0.0–41.7%
Guatemala	3	2	23	437	19	0.0–11.9%
Guyana	7	9	11	828	75	0.0–6.6%
Honduras	2	1	15	394	26	0.0–13.0%
Mexico	10	8	87	3,485	40	0.0–1.6%
Nicaragua	2	0	27	658	24	0.0–8.2%
Paraguay	0	0	5	115	23	0.0–35.5%
Suriname	325	448	564	61,402	109	0.0–0.1%
Venezuela	29	14	71	3,981	56	0.0–1.4%
Tour^c^	-	-	92	726	8	0.0–7.4%
Total	472	553	1,185^d^	92,324	78	0.0–0.1%

^a^ Residence or travel for a continuous period of at least 6 months

^b^ Retrospective incidence (RI) of *T*. *cruzi* infection was determined to be zero; 95% CIs are shown

^c^ Tour = consecutive stays in multiple CECs for a continuous period of at least 6 months in total

^d^ Reported by 1,121 donors, 51 of whom reported two or more long-term stays in various countries

**Fig 1:**
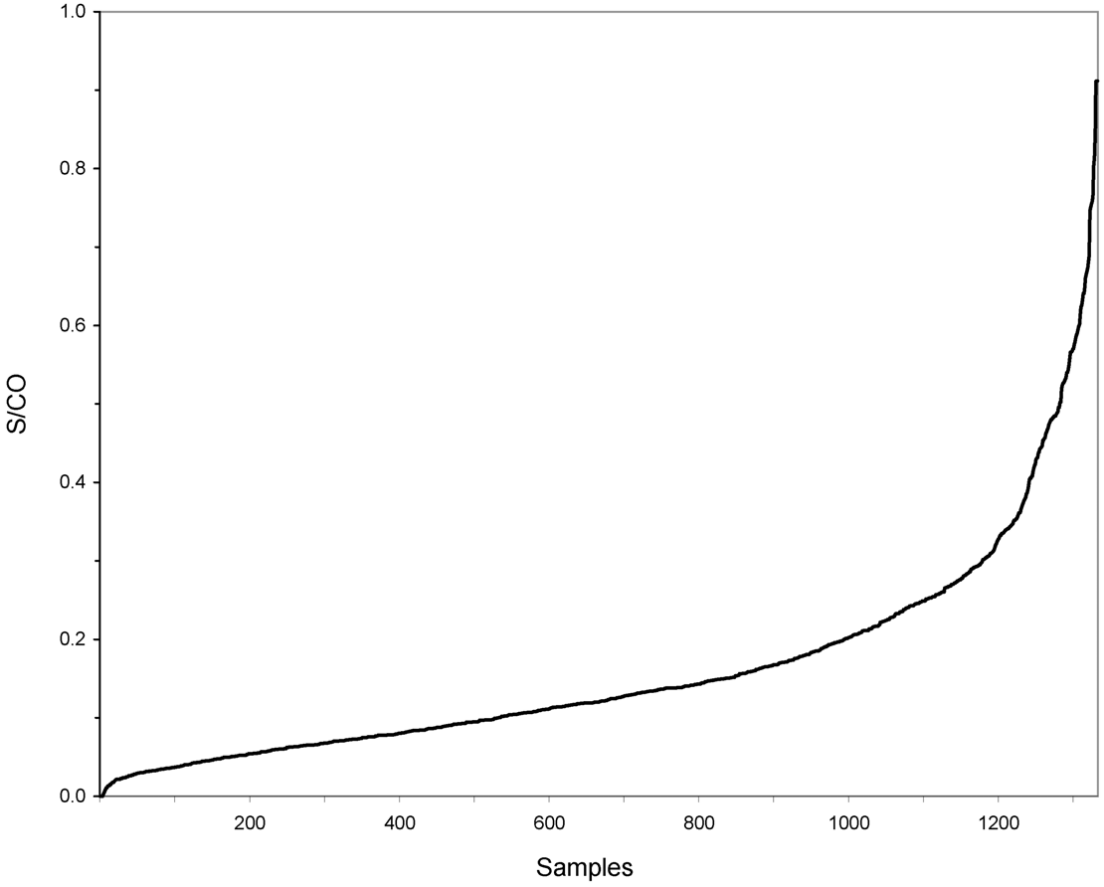
Distribution of EIA signals in serum samples screened for presence of IgG antibodies to *Trypanosoma cruzi*. The samples were collected from 1,333 Dutch blood donors reporting risk factors for *T*. *cruzi* infection. A sample-to-cutoff (S/CO) value < 1.0 is considered negative, indicating the absence of *T*. *cruzi* antibodies.

Regarding the 1,121 donors who reported long-term stays in endemic countries, the average length of stay was 78 months per donor. The donors resided for a total period of 92,324 months in CECs, which equals 7,694 years or 2.8 million overnight stays. Of those, 1.9 million (66.5%) and 0.4 million (15.8%) nights were spent in Suriname and Brazil, respectively. None of the 1,333 donors (0.0%; 95%, CI 0.0–0.4%) was seroreactive for IgG antibodies to *T*. *cruzi* (Fig 1), resulting in an overall retrospective incidence of 0.0% (95%, CI 0.0–0.1%) per year of residence in CECs.

## Discussion

This study is the first to examine the impact of long-term visits of blood donors to Latin American countries where *T*. *cruzi* infection is endemic. To assess whether specific blood safety measures are warranted in the Netherlands, risk factors for and the seroprevalence of *T*. *cruzi* infection were determined nation-wide in Dutch blood donors. More than 2.8 million overnight stays in endemic countries were reported, of which 1.9 million were spent in Suriname. A total of 472 donors were born in a country endemic for Chagas disease, while 553 donors reported having a mother who had been born in such a country. Surprisingly, not a single at-risk donor tested positive for *T*. *cruzi* antibodies. Cases of subclinical Chagas disease among at-risk donors in the Netherlands are thus extremely rare.

Most Latin American immigrants in the Netherlands come from Suriname, a country in the northeastern part of South America with approximately 566,000 residents, and Brazil, the largest country in the Latin American region with more than 200 million residents. According to the Dutch Bureau for Statistics, a total of 182,000 Surinamese and 13,000 Brazilian first-generation immigrants resided in the Netherlands in 2013. In addition, 166,000 Surinamese and 8,000 Brazilian second-generation immigrants were registered. According to the PAHO, the prevalence of *T*. *cruzi* infection in Suriname and Brazil is 1.29% and 1.02%, respectively, which is substantially lower compared with Bolivia, Argentina, El Salvador, and Honduras. Those countries are hyperendemic for Chagas disease, with estimated prevalences of 6.75%, 4.13%, 3.37%, and 3.05%, respectively [14]. This study demonstrates that only a small number of Dutch blood donors has roots in these endemic countries. Of course this cannot automatically be extrapolated to donor populations in other non-endemic countries as immigrant populations vary from country to country [13,17].

Laboratory diagnosis of chronic *T*. *cruzi* infection is challenging. The direct detection of parasites is generally difficult, even with polymerase chain reaction techniques, due to low levels of parasitemia in the chronic phase of infection. The laboratory diagnosis in blood donors and in patients is primarily based on serology. Unfortunately, available serology-based assays often suffer from suboptimal specificity and/or sensitivity. In general, enzyme immunoassays perform better than assays based on other formats, such as indirect hemagglutination assays or particle agglutination assays [18] and polymerase chain reaction [19]. Only a few assays meet the requirements for single-assay screening of blood donations. For this study we used the *T*. *cruzi* EIA of Ortho Clinical Diagnostics on the basis of excellent performance characteristics [10,15,16]. Having a reported sensitivity of 97.7–100% it is unlikely that *T*. *cruzi* infected donors were not detected by this assay.

No serologic signs for *T*. *cruzi* infection were found among 325 donors born in Suriname (95% CI, 0.0%-1.5%), 448 donors having a Surinamese mother and 564 donors reporting one or more long-term stays in Suriname (including 1.9 million overnights stays). This suggests that the prevalence of *T*. *cruzi* infection among immigrant donors from Suriname is below the PAHO estimate of 1.29% for Surinamese residents [14]. Given the absence of *T*. *cruzi* infection among all at-risk donors in this study, the PAHO estimates for local Chagas prevalences may be of limited value for estimating the risk among immigrant donors in non-endemic countries. In general, this probably also applies to blood donors from non-endemic countries whilst visiting Latin America.

*T*. *cruzi* exists as extracellular forms in the blood and is susceptible to processing and storage conditions. Leukoreduction by centrifugation or filtration reduces *T*. *cruzi* infectivity [20,21]. Martin and co-workers showed the ability of *T*. *cruzi* to survive in blood during storage at +4 or -80°C [22]; not all, but the vast majority of viable *T*. *cruzi* parasites died during long-term cold storage, which suggests a residual potential for transmission by transfusion. There is, however, no evidence that recipients of red blood cells and frozen blood products acquire *T*. *cruzi* infection; transfusion-associated transmission in developed countries outside Latin America appears to be restricted to platelet components stored at room temperature [6,23–25].

Molecular genetic analysis has identified six lineages, TcI to TcVI, with specific geographical distribution [26]. Different lineages of *T*. *cruzi* are associated with varying severity of disease [27], and may pose varying risks of transmission as well. The rate of transmission by blood products from serologically confirmed positive donors originating from Mexico and countries in Central America, where TcI circulates, is low [28]. Higher levels of parasitemia seem to be typical for TcII to TcVI [27]; these lineages predominantly circulate in the central and southern parts of South America. The higher levels of parasitemia may be the explanation that for example in Spain—having historical links to central and southern parts of South America—transmission by transfusion is observed, while in the Netherlands, with historical links limited to Suriname and the Caribbean, blood-borne transmission of *T*. *cruzi* seems absent.

Vertical transmission is the most important mode of transmission of *T*. *cruzi* in non-endemic areas [29,30]. In Spain, the overall *T*. *cruzi* seroprevalence in 1,975 pregnant women from 16 Chagas-endemic countries was 11.4% in one study [31]. In another Spanish study 4.0% of 3,839 pregnant women from Latin America were found seropositive [32]. The vertical transmission rates in these studies were 3.7% and 2.6%, respectively. Congenital transmission rates of 5.0% and 2.7% in endemic versus non-endemic countries were found in a study by Howard and colleagues [33], providing further indication that donors who are born to infected mothers may pose a threat to blood safety if they are not tested for *T*. *cruzi* infection. However, to date no transfusion-transmitted cases are documented in which donors who were born outside of Latin America were involved. This might be partially due to screening programs and deferral policies for such donors being in place in several non-endemic countries, but it can also be imputed to the limited potential of *T*. *cruzi* to be transmitted by cold-stored blood products, such as red blood cells and fresh frozen plasma.

In conclusion, asymptomatic *T*. *cruzi* infection appears to be extremely rare in the Dutch blood donor pool, and may even be absent. This is not only reassuring in terms of blood safety in the Netherlands, it also suggests a prevalence of *T*. *cruzi* infection among immigrant donors from Latin America below PAHO estimates for Latin American residents. At present, blood safety interventions to mitigate the risk of *T*. *cruzi* transmission by blood products in the Netherlands would be highly cost-ineffective and are thus not required. This may apply to other non-endemic countries as well, in particular to countries with low numbers of immigrant donors from Latin American countries with the highest prevalence of Chagas disease.

## Abbreviations

CEC: country endemic for Chagas disease
EIA: enzyme immuno assay
IgG: immunoglobulin G
PAHO: Pan American Health Organization
